# Machine learning models for predicting survival in lung cancer patients undergoing microwave ablation

**DOI:** 10.3389/fmed.2025.1561083

**Published:** 2025-05-07

**Authors:** Yufan Liu, Zihang Wang, Xiaowen Cao, Miaoyan Liu, Lou Zhong

**Affiliations:** Department of Thoracic Surgery, Affiliated Hospital of Nantong University, Nantong, Jiangsu, China

**Keywords:** lung cancer, machine learning, microwave ablation, survival, prognosis, predict model

## Abstract

**Objective:**

To develop and validate predictive models assessing survival outcomes in patients with non-small cell lung cancer (NSCLC) treated with microwave ablation (MWA), enabling clinical decision support and personalized care.

**Methods:**

This retrospective study analyzed data from 181 NSCLC patients who underwent MWA between May 2013 and May 2023. Prognostic factors were identified through univariate analysis, and predictive models were constructed using machine learning techniques. The model validation was conducted using cross-validation to ensure the model’s robustness and generalizability.

**Results:**

Univariate analysis revealed several significant prognostic factors, including tumor stage, serum phosphorus levels, patient age, average hemoglobin levels, ground-glass opacities (GGO), and pleural traction. The presence of GGO and pleural traction was associated with worse prognosis, and these factors were incorporated into the model. After training, the best-performing model achieved an area under the curve (AUC) of 0.742, demonstrating a good balance between sensitivity and specificity. Cross-validation and external validation further confirmed the robustness and generalizability of the model, with similar AUC values observed in both validation cohorts. The model effectively predicted the 1-, 3-, and 5-year survival rates for NSCLC patients treated with MWA. These findings suggest that the model can serve as a reliable tool for clinical decision-making and support individualized treatment strategies.

**Conclusion:**

The developed predictive model effectively assesses prognosis in NSCLC patients treated with MWA, supporting individualized treatment strategies and improving clinical decision-making.

## Introduction

Lung cancer is the leading cause of cancer-related mortality globally, with non-small cell lung cancer (NSCLC) accounting for the majority of cases (1). Lung cancer ranks first in both incidence and mortality in China (2). While surgical resection remains the standard treatment, many patients are ineligible due to medical contraindications, necessitating alternative treatments such as microwave ablation (MWA) (3, 4).

Ablation techniques including MWA, RFA, and cryoablation are widely used in the medical field (5, 6). MWA is a minimally invasive technique that utilizes high-frequency microwave energy to induce thermal necrosis in tumor tissues (7). Its advantages include reduced surgical trauma, shorter recovery time, and preservation of surrounding healthy tissues, making it a valuable option for patient ineligible for surgery. However, while short-term benefits of MWA are well-documented, its impact on long-term survival remains unclear, necessitating further investigation.

Radiomics is an emerging multidisciplinary field that combines medical imaging, computer science, and bioinformatics to extract a large number of quantitative features from medical imaging data. These features can reflect information about tissue morphology, texture, and intensity, and can be used for disease diagnosis, prognosis, and treatment evaluation. The basic process of radiomics includes image acquisition, image processing, feature extraction, and data analysis. High-quality imaging data is obtained through imaging techniques such as CT, MRI, and PET, followed by preprocessing to extract regions of interest, from which quantitative features are extracted. Machine learning and data mining techniques are then employed to analyze these features, revealing patterns and relationships associated with clinical outcomes. Radiomics has broad application prospects in oncology, as it can predict the biological behavior of tumors, treatment response, and patient prognosis. It can also be used in the study of other diseases, such as cardiovascular and neurological disorders. Despite significant progress, radiomics still faces challenges such as data standardization, optimization of feature extraction methods, and integration of multicenter data. With the continuous development of technology and interdisciplinary collaboration, radiomics is expected to play an increasingly important role in medical research and clinical applications (8).

Recent advancements in machine learning (ML) have revolutionized medical research, enabling the integration of structured data, such as clinical parameters, and unstructured data, including imaging features, for predictive modeling (9). Driven by the wave of digitization, the rapid progress of artificial intelligence, especially in tools related to machine learning and data mining, allows doctors to make decisions more swiftly (10). By leveraging these technologies, this study aims to address the unmet need for accurate prognostic tools in NSCLC patients undergoing MWA.

This study uniquely integrates structured clinical data and machine learning techniques to develop a predictive model for NSCLC prognosis following MWA, providing insights into patient-specific risk factors and supporting clinical decisions (11).

## Materials and methods

### Sequence chart

**FIGURE 1:**
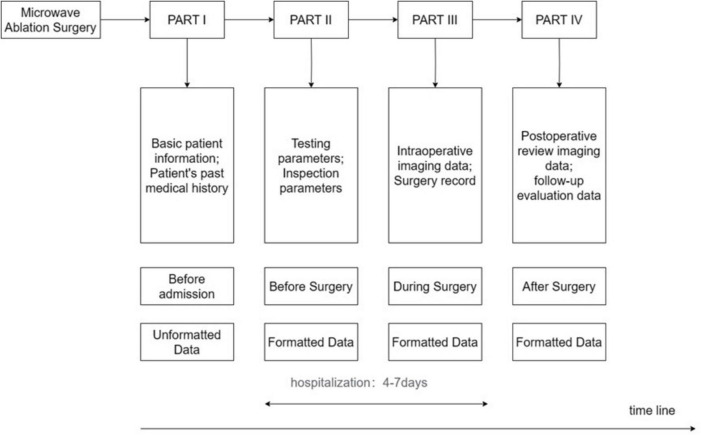
Sequence chart of MWA PART.

In our research, we used a timeline-based sequence chart to divide the ablation process into four parts: PART I involves pre-admission information, including non-structured data such as patient demographics and medical history; PART II covers the preoperative phase, encompassing structured data such as preoperative blood tests and imaging examinations; PART III focuses on the intraoperative phase, including structured data such as intraoperative imaging and surgical plans; and PART IV addresses the postoperative phase, gathering structured data such as postoperative review imaging and follow-up assessments, including survival data. PARTs II and III represent the hospitalization phase, with a typical hospital stay of 4–7 days. This study aims to utilize artificial intelligence technology to construct a comprehensive predictive model by analyzing EMRs collected throughout the inpatient process. Figure 1 provides a detailed depiction of these PARTs, including the types of data involved in each PART and their placement within the entire ablation process.

### Model development

In terms of data preprocessing, we conducted preprocessing steps such as handling missing values and standardizing structured data. For unstructured data, we employed NER technology to extract crucial factors, enhancing their integration into the model. The dataset was divided into training and testing sets to ensure the effectiveness and generalization of the model. Regarding feature engineering, we identified key factors related to patient prognosis through data mining at each PART.

We utilized constructed temporal graph models for modeling, predicting IV separately using I, II, III, and combining I+II, I+III, II+III, I+II+III. Seven different machine learning models were created for 10-fold cross-validation, employing a multi-model comparison approach. Comprehensive evaluations of each model were conducted through ten-fold cross-validation and ROC curves, providing a deeper understanding for survival prediction in the entire process of MWA for lung cancer. Compared to single-PART models, the combined prediction of multiple models demonstrated superior overall performance, possibly reflecting the synergistic effect of information from different PARTs, offering richer information for improving survival outcome prediction.

Based on these factors, we applied machine learning to train the seven different models. After training, model parameters were optimized to enhance performance. This process ensured that the experience gained and optimization results during model construction were thoroughly considered.

### Patient criteria

**FIGURE 2:**
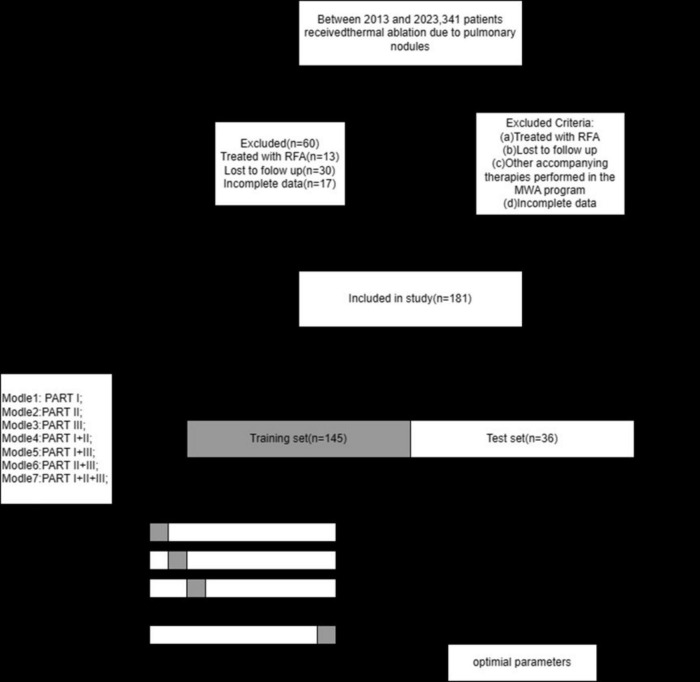
Flowchart of this study.

Figure 2 illustrates the selection of patients who underwent microwave ablation treatment at the Affiliated Hospital of Nantong University between May 2013 and May 2023. Patients were randomly assigned to training or validation groups in a 4:1 ratio. A total of 341 patients were included in the study, excluding 13 patients treated with radiofrequency ablation, 30 patients lost to follow-up, and 17 patients with incomplete data, resulting in the inclusion of 181 patients in the model. These 181 patients were randomly selected in a 4:1 ratio, with 145 patients included in the training group and 36 patients in the validation group. Subsequently, seven different models were constructed for the training group based on temporal graph selection criteria. Table 1 shows the selection criteria, each model underwent four machine learning ten-fold validations, and the model with the highest accuracy was selected and validated using the validation set. Figure 1 shows the validation process. Table 2 describes the baseline data of the patients, including basic characteristics and clinical information for both the training and validation set.

**TABLE 1:** Model-outcome prediction matrix.

	Predict data	Predict outcome
Model 1	PART I	PART IV
Model 2	PART II	PART IV
Model 3	PART III	PART IV
Model 4	PART I+II	PART IV
Model 5	PART I+III	PART IV
Model 6	PART II+III	PART IV
Model 7	PART I+II+III	PART IV

**TABLE 2:** Patient demographics and baseline characteristics.

Variables	Overall (181)	Training cohort (145)	Validation cohort (36)	*p*-value
Age (y)				<0.001
<60	29	22	7	
>60	152	114	38	
Gender				
Male	117	87	30	
Female	64	48	16	
Tumor stage				<0.001
I	117	87	30	
II	48	36	12	
III	30	23	7	
IV	5	4	1	
Tumor diameter (cm)				0.362
<3	16	12	4	
>3	165	124	41	
Treatment numbers				<0.01
1	169	127	42	
>1	12	9	3	
Underlying pulmonary diseases				
Pneumothorax	21	16	5	
Pleural effusion	37	31	9	
MCH	83	47	16	<0.01
Serum phosphorus	45	34	11	<0.01
Tumor position				0.665
Upper left lobe	46	34	12	
Left lower lobe	38	29	9	
Right upper lobe	60	45	15	
Right middle lobe	5	4	1	
Right lower lobe	42	32	10	
Atrial fibrillation	4	3	1	0.017
GGO	23	16	7	0.247
Pleural retraction	11	8	3	0.058
Mortality	118	88	30	
Median OS (months)	21.94			

NSCLC tumor staging was determined based on the 8th edition of the clinical TNM staging system by the International Union for Cancer Control (UICC). All NSCLC patients underwent chest CT scans before MWA surgery to assess tumor location, quantity, and size. All laboratory tests were conducted 4 days before MWA.

### Evaluation

The primary outcome measures include the patient’s survival status and survival time, defined as the interval from the initiation of MWA to death or the last follow-up. For patients who passed away during the follow-up, OS is calculated as the interval from the MWA procedure to the date of death. For patients who are still alive or lost to follow-up, OS is calculated as the interval from the MWA procedure to the date of the last follow-up. In cases where patients have neither died nor progressed, the review date is defined as the date of the last clinical assessment. The patient’s survival status is determined during the regular follow-up in May 2023, reflecting the patient’s survival status at that specific time.

### Procedure and peripheral management

The indications and procedures for MWA adhere to the standards set by CIRSE and are implemented by experienced interventional radiologists. The MTC-3C MWA system (Vision Medicine) is utilized for MWA, with a microwave emission frequency of 2450 ± 30 MHz and adjustable continuous wave output power ranging from 5 to 120 W. The MWA needle has an effective length of 10–18 cm, an outer diameter of 13–17 g, and an effective tip of 15 mm. Preoperative CT provides necessary information for treatment planning, including identification of suitable ablation sites, puncture site locations, optimal puncture trajectories, and the required number of MWA needles. Local anesthesia or intravenous anesthesia is administered as needed. The needles are introduced to the planned sites, and their positions are confirmed via CT. Subsequently, MWA is performed according to the planned power and duration, with adjustments made if necessary. The procedure is terminated when the ablation zone exhibits a 5–10 mm margin. MWA parameters are selected based on the recommended production ablation zone of the MWA system. Finally, repeat chest CT scans are conducted to assess the ablation zone and detect potential complications. Follow-up MWA visits are scheduled 1–5 days post-hospitalization, with the first follow-up at 3–4 weeks, followed by subsequent visits every 3 months. These follow-ups include physical examinations and chest CT or PET/CT scans to evaluate treatment efficacy and detect recurrence or distant metastasis.

### Natural language processing

In this study, we utilized NER technology to extract relevant medical history and symptoms from unstructured data in patients’ EMRs. The NER, implemented in Python (version 3.7), employed the LERT-BiLSTM-CRF model, integrating LERT pre-trained models, BiLSTM, and CRF layers. LERT utilized a language-informed pre-training strategy on three types of language features (word position in the sentence, NER labels, word part-of-speech) for pre-training tasks (12). BiLSTM further utilized LERT’s vector representation to extract richer contextual features. The CRF layer utilized the contextual features extracted by BiLSTM to determine the label for each word. To enhance training efficiency, we divided excessively long sentences in electronic medical records, averaging 128 characters per sentence, and conducted masked language model training tasks. NER experiments on EMRs from five hospitals considered variations in format and lack of standardization, addressing these through punctuation-based sentence segmentation for standardized input. The current average F1 score for the tested EMRs is 90.67%, with an accuracy of 94.44% (13).

### Integration of EMR and radiomics

Integrating EMR and radiomics provides complementary information for more accurate prognosis prediction. Radiomics parameters, such as standard deviation and inertia, quantify tumor heterogeneity and texture features, revealing microstructural changes; for instance, higher standard deviation often correlates with ineffective treatments, while lower inertia values post-treatment suggest successful tumor ablation. Meanwhile, EMRs provide detailed patient histories, treatment records, lab results, and follow-up data, offering a macroscopic view of disease progression and treatment response. This study demonstrates that larger tumor sizes and shallower GGO margins are linked to ineffective treatments, highlighting the importance of combining clinical and imaging data. By integrating radiomics features with EMR data, predictive models can dynamically monitor treatment effects, validate imaging biomarkers, and guide personalized strategies, improving survival rates and quality of life.

### Statistical analysis and machine learning

Four machine learning algorithms—logistic regression (LR), random forest (RF), support vector machine (SVM), and extreme gradient boosting (XGB)—were employed to develop predictive models. Seven models were constructed using combinations of input data (e.g., pre-operative factors, intra-operative factors). Each model was trained and validated using 10-fold cross-validation.

In this experiment, we utilized SPSS 25.0 for Windows. Categorical variables were described as frequencies and percentages, while continuous variables were described as SD ± median/mean. Continuous variables were compared using Student’s *t*-test or Mann-Whitney U test, and categorical variables were compared using the chi-square test between the two cohorts. Univariate analysis of potential predictors for patient survival was conducted in the training cohort, with variables having *p* < 0.05 in the univariate analysis inputted as candidate variables into the multivariate Cox regression analysis. In the multivariate analysis, variables with *p* < 0.05 were considered statistically significant. The results of the multivariate analysis were presented using R (R version 4.3.1).

## Results

### Patient characteristics and clinical outcomes

The characteristics of the enrolled patients are presented in Table 1, comprising a total of 181 individuals (64 females, 177 males; mean age: 54.5 years ± 34.5 years). The average follow-up duration was 30.5 months. Factors such as age, tumor stage, serum phosphorus, atrial fibrillation, and average hemoglobin levels exhibited significant differences among baseline characteristics.

### Selection of radiomics factors

Based on relevant literature, we selected GGO, vascular invasion, and pleural retraction as key features for our study. These features hold significant clinical importance in various lung diseases, particularly lung cancer. GGO appears on CT images as a localized haziness that does not obscure the underlying pulmonary vessels and bronchial structures. It is closely associated with the occurrence of lung cancer, especially early-PART adenocarcinoma. The presence and characteristics of GGO can help assess the malignancy and progression of lesions (14). Pleural retraction refers to the localized indentation or deformation of the pleura due to tumor or lesion pulling or adhesion. This feature is significant in assessing local tumor invasiveness and is often linked to higher tumor stages and poorer prognosis (15). The selection of these features is based on their high relevance and clinical importance documented in the literature. They not only aid in more accurate evaluation and diagnosis of lung lesions but also provide critical prognostic information, helping to develop personalized treatment plans. Analyzing these features comprehensively is expected to enhance the predictive performance of the model, thereby improving patient management and treatment in clinical practice.

### Predictive factors for the prognostic model

In constructing the final prognostic prediction model, key factors were incorporated, including tumor stage (T), patient age, serum phosphate levels, and mean corpuscular hemoglobin (MCH). Additionally, three critical imaging and clinical features—GGO and pleural retraction—were selected due to their significant impact on patient prognosis and their contribution to enhancing the model’s predictive accuracy. Atrial fibrillation, present in only four patients (2.2% of the total), was excluded from the analysis. The final model includes seven predictive factors, categorized based on the different PARTs they belong to. Factors in PART I are age and tumor stage, PART II includes average hemoglobin and serum phosphorus, and PART III accounts for the number of puncture surgeries performed during the operation. All relevant factors and their respective time points are described in Table 3.

**TABLE 3:** Prognostic model predictors.

PART	Type of data	Time point	Factors
Part I	Unformatted data	Before admission	Patient demographics
			Patient tumor stage
Part II	Formatted data	Before MWA	Testing parameters
			Inspection parameters
Part III	Formatted data	During MWA	Puncture times
			Surgical site
			Surgery time
Part IV	Formatted data	After MWA	Survival status
			Survival time

### Machine learning model validation results

**FIGURE 3:**
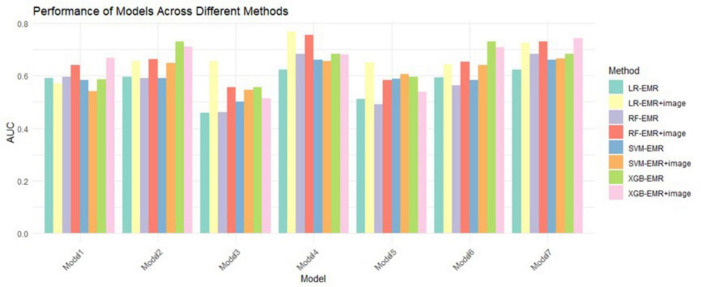
10-fold CV mean AUC of 4 ML algorithms. LR, logistic regression; RF, random forest; XGB, extreme gradient boosting; SVM, support vector machines.

Based on significant risk factors identified through multivariate Cox regression analysis on the training cohort, four machine learning algorithms—LR, RF, SVM, and XGB—were employed to construct predictive models. These models were evaluated using ten-fold cross-validation, and their performance was compared through ROC curve analysis and AUC values, with higher AUC values indicating better accuracy in predicting patient survival outcomes. The evaluation results are illustrated in Figure 3.

Among all the models, Model 7 demonstrated the highest AUC of 0.716, indicating its strong performance in predicting prognosis risk for patients with NSCLC undergoing MWA treatment. This model was selected for its superior accuracy in survival prediction.

In constructing the final prognostic prediction model, the following key factors were incorporated: T, patient age, serum phosphate levels, and MCH. Additionally, two critical imaging and clinical features—GGO and pleural retraction—were included due to their significant impact on patient prognosis and contribution to enhancing the model’s predictive accuracy. Table 4 provides a detailed overview of these factors and their contributions to the final model.

**TABLE 4:** Mean AUC of 10-fold cross validation with 4 ML algorithms in 7 prediction models.

Model	Model 1	Model 2	Model 3	Model 4	Model 5	Model 6	Model 7
LR-EMR	0.591	0.595	0.458	0.624	0.512	0.593	0.624
LR-EMR+Img	0.572	0.657	**0.657**	**0.767**	0.651	0.643	0.727
RF-EMR	0.597	0.591	0.461	0.683	0.492	0.564	0.683
RF-EMR+Img	0.641	0.663	0.555	0.756	0.584	0.653	0.731
SVM-EMR	0.584	0.592	0.500	0.661	0.589	0.583	0.661
SVM-EMR+Img	0.541	0.649	0.547	0.656	**0.670**	0.642	0.665
XGB-EMR	0.586	**0.732**	0.556	0.684	0.597	0.732	0.684
XGB-EMR+Img	**0.669**	0.712	0.513	0.680	0.539	**0.790**	**0.742**
Mean-auc- EMR	0.589	0.627	0.493	0.663	0.547	0.618	0.663
Mean-auc- EMR+Img	0.657	0.672	0.568	0.714	0.611	0.682	0.716

Bold values represent the highest AUC, while underlined values represent the lowest AUC.

### Model performance

Among the four machine learning models, the XGB model demonstrated the best performance, achieving an AUC of 0.742 in the validation cohort, indicating superior predictive accuracy. Table 5 provides a detailed comparison of the models’ AUC values and performance metrics.

**TABLE 5:** Mean AUC of 10-fold cross validation for 4 machine learning algorithms in 7 prediction models.

Model	AUC (training)	AUC (validation)
Logistic regression	0.727	0.715
Random forest	0.731	0.783
Support vector machine	0.665	0.552
XGBoost	0.742	0.711

### Calibration curve for model validation

**FIGURE 4:**
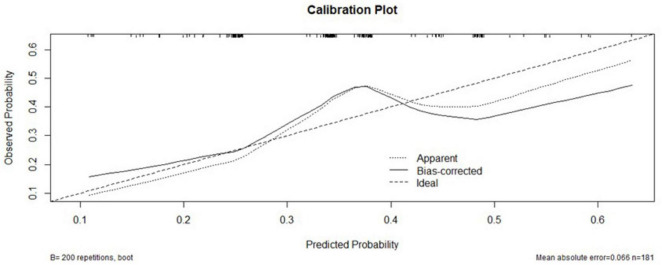
Calibration curve for OS prediction after MWA.

The XGBoost model calibration curve (Figure 4) demonstrated excellent agreement between predicted and observed probabilities, with MAE = 0.066 and MSE = 0.0067. Feature importance analysis revealed tumor stage and serum phosphorus levels as the most critical predictors, followed by patient age and average hemoglobin levels.

### Patient feature importance ranking

**FIGURE 5:**
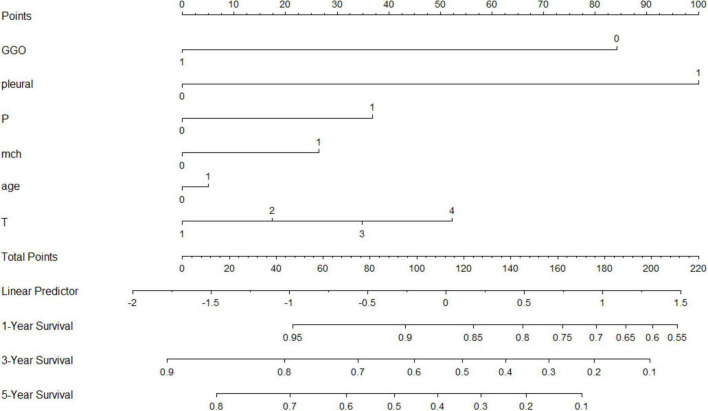
Nomogram used to predict OS after MWA.

Additionally, we constructed a feature importance ranking using the XGB algorithm. Based on Figure 5, we selected predictive factors deemed valuable. The importance scores of features reflect their significance and contribution within the model. Features with higher importance scores are considered to have a greater impact on the model’s predictions and should be prioritized for further in-depth research and analysis to understand their relationship with the target variable. Conversely, features with lower importance scores have a lesser impact on the model results and may be considered for exclusion during model training or feature selection processes, potentially simplifying the model without significantly reducing its predictive performance.

Based on the feature importance ranking shown in the diagram, the importance of each feature decreases sequentially within the model. The ranking reflects the contribution of each feature to the model’s predictions, with higher-ranked features having a more significant impact and thus warranting more attention in further research, while lower-ranked features might be considered for exclusion to simplify the model.

### Nomogram and Kaplan-Meier analysis

**FIGURE 6:**
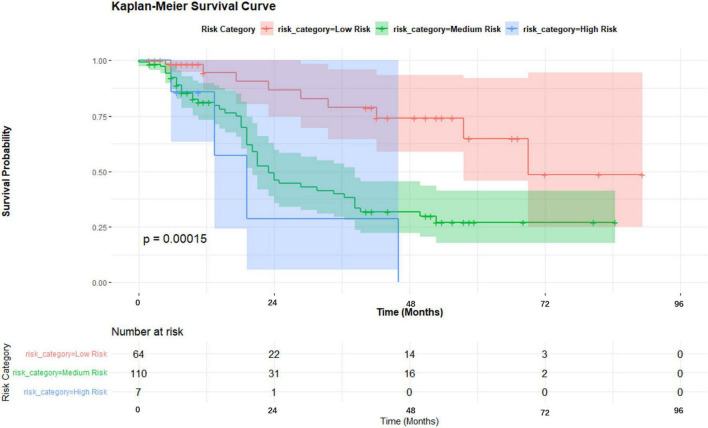
Kaplan-Meier survival curves by risk category.

A nomogram was developed incorporating tumor stage, serum phosphorus levels, age, and hemoglobin levels to predict survival probabilities (Figure 5). Risk stratification based on the nomogram divided patients into low-risk, medium-risk, and high-risk groups. Kaplan-Meier survival curves showed significant differences in survival among these groups (*p* < 0.001), with the high-risk group exhibiting the poorest survival outcomes (Figure 6).

### Clinical implications

**FIGURE 7:**
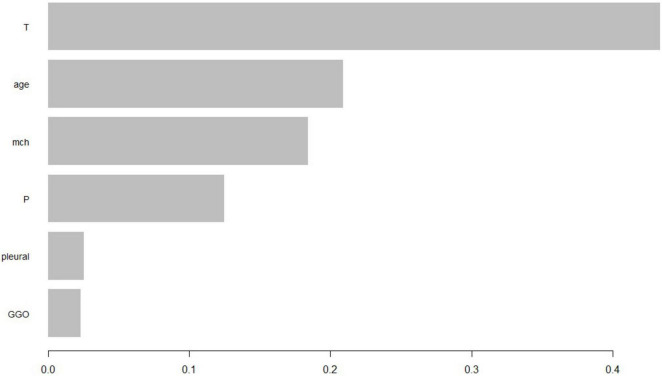
The importance ranking of different factors.

The findings suggest that the XGB-based predictive model and nomogram are robust tools for assessing prognosis in NSCLC patients undergoing MWA. These tools can support personalized treatment planning and improve patient management (Figure 7).

## Discussion

Microwave ablation has become an important treatment modality for NSCLC due to its minimally invasive nature and efficacy in tumor control. By creating a thermal necrotic zone that encompasses the tumor with a safety margin, MWA ensures effective tumor destruction while minimizing damage to surrounding tissue. Prior studies have demonstrated favorable survival outcomes with MWA, making it a viable alternative for patients ineligible for surgery due to tumor location or comorbidities (16).

Tumor staging remains a cornerstone in determining the prognosis of NSCLC patients.By assessing key factors such as tumor size, extent of local invasion, and distant metastasis, the staging system provides important information regarding the severity and potential progression of the disease. It plays a crucial role in guiding treatment decisions and predicting patient outcomes.

**FIGURE 8:**
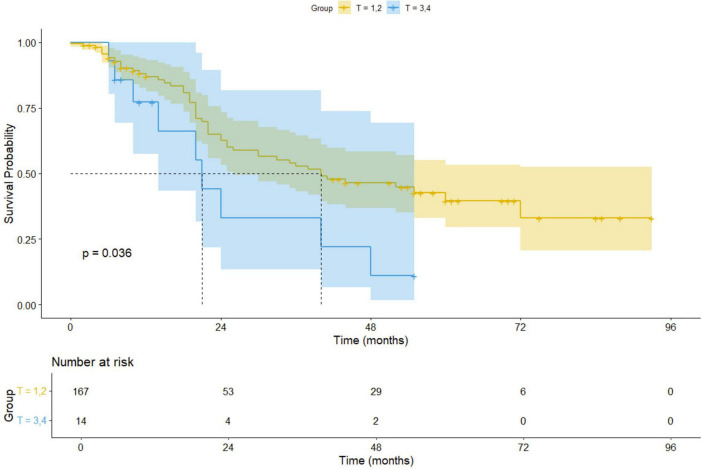
Kaplan-Meier survival curves for early-stage and advanced-stage NSCLC patients.

For early-stage (I–II) non-small cell lung cancer (NSCLC) patients, surgical treatment, including procedures such as lung lobe resection and lymph node excision, remains the preferred approach, offering high long-term survival rates (17). However, for patients with advanced (III–IV) or inoperable tumors, microwave ablation (MWA) combined with radiation therapy provides a promising alternative. Our Kaplan-Meier survival analysis results indicate a significant difference in survival outcomes between early-stage (I–II) and advanced-stage (III–IV) patients undergoing MWA treatment (*p* = 0.036), suggesting that disease stage plays an important role in prognostic evaluation. Figure 8 displays the Kaplan-Meier survival curves, comparing the survival of early-stage and advanced-stage NSCLC patients. These curves clearly illustrate the survival differences between the two groups, further supporting our analysis results.

Moreover, MWA offers distinct advantages for specific patient populations. For elderly patients or those with severe cardiopulmonary dysfunction, surgical treatment may pose higher perioperative risks. In contrast, MWA, as a minimally invasive therapy, presents fewer complications, quicker recovery, and lower risks compared to other localized treatments like radiofrequency ablation (RFA). Future studies evaluating the efficacy of MWA in treating NSCLC should involve larger-scale prospective trials to optimize patient stratification strategies and improve personalized treatment approaches.

Tumor staging alone, while crucial, does not fully account for all variables that influence the prognosis of NSCLC patients. Various other factors, such as serum phosphorus levels, age, and hemoglobin concentration, play significant roles in shaping patient outcomes.

Elevated circulating phosphorus has been implicated in cancer pathogenesis through its role in promoting cell proliferation, angiogenesis, and chromosomal instability. Research has shown that high phosphorus levels correlate with an increased risk of developing lung cancer and poorer survival outcomes. This emphasizes the potential role of serum phosphorus as a prognostic marker in the management of NSCLC (18).

Age is a fundamental factor in assessing the overall health and prognosis of lung cancer patients. As patients age, the decline in physiological function, immune responsiveness, and the ability to recover from injury can worsen disease outcomes. Studies have consistently shown a strong association between advancing age and both higher incidence and poorer prognosis of lung cancer. Older patients often face additional challenges, including a reduced ability to tolerate aggressive therapies and an increased risk of comorbidities, which further complicates treatment and impacts survival (19).

Hemoglobin is critical for oxygen transport, and its role in cancer prognosis cannot be overstated. Low hemoglobin levels, indicative of anemia, are associated with poorer tumor oxygenation, impaired immune function, and reduced metabolic capacity. These factors contribute to a diminished ability to respond to therapies such as ablation. Studies have found that lung cancer patients with anemia have worse survival rates following ablation treatment, underscoring the importance of maintaining optimal hemoglobin levels for better therapeutic outcomes (20).

Imaging plays an indispensable role in assessing lung cancer. Ground-glass opacities (GGOs), often identified on chest CT scans, represent areas of early tumor infiltration or inflammatory change. GGOs are typically associated with less aggressive forms of lung cancer, and patients with GGOs tend to have better survival outcomes compared to those with solid nodules. However, the persistence or progression of GGOs can indicate tumor progression, necessitating more aggressive treatment and careful monitoring (21, 22).

Pleural retraction, another radiological finding, indicates that the tumor has invaded or adhered to the pleural surface, often complicating treatment. Tumors exhibiting pleural retraction are usually indicative of advanced disease and may require more extensive surgical resection or multimodal therapies. Furthermore, the presence of pleural retraction may impair respiratory function, directly affecting a patient’s quality of life and prognosis (22).

GGOs are radiological findings seen on chest CT scans, characterized by hazy areas of increased attenuation that do not obscure underlying structures. GGOs often represent areas of early tumor infiltration or inflammatory changes and can be associated with both benign and malignant conditions. In the context of lung cancer, the presence and extent of GGOs can provide insights into the tumor’s biological behavior. Studies have shown that patients with GGOs in their imaging may have a better prognosis compared to those with solid nodules, as GGOs often indicate a less aggressive form of cancer. However, the persistence or growth of GGOs can signal tumor progression, necessitating careful monitoring and potentially more intensive treatment strategies.

Pleural Retraction refers to the pulling in or distortion of the pleural surface caused by tumor invasion or fibrosis. This sign on imaging can indicate significant local disease progression or adherence of the tumor to the pleura. The presence of pleural retraction is often associated with more advanced disease and can complicate surgical interventions. Tumors with associated pleural retraction may require more extensive surgical resection or multimodal treatments to manage the disease effectively. Additionally, pleural retraction can impact respiratory function and patient quality of life, highlighting the importance of addressing this factor in treatment planning and patient management.

Accurate prognosis in NSCLC requires an integrated approach beyond tumor staging alone. This study demonstrates that combining tumor staging with biochemical markers (serum phosphorus), age, hemoglobin levels, and radiological findings (GGO, pleural traction) enhances survival prediction for patients undergoing MWA. By capturing complex interactions among these factors, our machine learning-based model provides a more comprehensive assessment, improving individualized treatment strategies.

Machine learning enables the identification of intricate prognostic patterns, overcoming the limitations of single-factor analyses. Our model achieved robust predictive performance, validated internally and externally, reinforcing its clinical applicability. It allows for personalized risk assessment, helping clinicians optimize treatment decisions and resource allocation.

Despite promising results, limitations exist. The retrospective design introduces potential bias, and the single-institution dataset may limit generalizability. Additionally, the absence of long-term follow-up constrains assessment of prediction durability. Future studies should incorporate larger, multicenter cohorts with extended follow-up to validate the model’s reliability and applicability in broader clinical settings.

In conclusion, this study highlights the value of integrating multiple prognostic factors using machine learning to improve survival prediction in NSCLC patients treated with MWA. With further validation, this model holds potential as a clinical tool for personalized decision-making and optimized patient management.

## Data Availability

The datasets presented in this study can be found in online repositories. The names of the repository/repositories and accession number(s) can be found below: https://doi.org/10.4121/7285a143-eeaa-4ee8-91b1-e3ad10ed5b20.v1.
